# Mitochondrial oxidative stress or decreased autophagy in osteoblast lineage cells is not sufficient to mimic the deleterious effects of aging on bone mechanoresponsiveness

**DOI:** 10.18632/aging.206213

**Published:** 2025-03-18

**Authors:** Ana Resende-Coelho, Md Mohsin Ali, Alicen James, Aaron Warren, Landon Gatrell, Ilham Kadhim, Qiang Fu, Jinhu Xiong, Melda Onal, Maria Almeida

**Affiliations:** 1 Division of Endocrinology and Metabolism, University of Arkansas for Medical Sciences, Little Rock, AR 72205, USA; 2 Department of Physiology and Cell Biology, University of Arkansas for Medical Sciences, Little Rock, AR 72205, USA; 3 Department of Orthopaedic Surgery, University of Arkansas for Medical Sciences, Little Rock, AR 72205, USA; 4 Center for Musculoskeletal Disease Research, University of Arkansas for Medical Sciences, Little Rock, AR 72205, USA

**Keywords:** Atg7, tibia compressive loading, Sod2, Osx1-Cre, osteocytes

## Abstract

Exercise-induced mechanical load stimulates bone cells, including osteocytes, to promote bone formation. The bone response to loading is less effective with aging, but the cellular and molecular mechanisms responsible for the impaired mechanoresponsiveness remain unclear. Excessive mitochondrial reactive oxygen species (mtROS) and deficient autophagy are common aging mechanisms implicated in decreased bone formation in old mice. Here, we confirmed that the osteogenic effects of tibia compressive loading are lower in old versus young female mice. We also examined whether an increase in mtROS or decreased autophagy in osteoblast-lineage cells of adult female mice could mimic the deleterious effects of aging. To this end, we loaded mice lacking the antioxidant enzyme superoxide dismutase 2 (*Sod2*) or autophagy-related 7 (*Atg7*) in cells targeted by Osterix1 (Osx1)-Cre. Osteocytes in *Atg7^Δ^*^Osx1^ exhibited altered morphology and decreased osteocyte dendrite projections. Two weeks of loading increased cortical bone mass and bone formation rate at both periosteal and endosteal surfaces of Osx1-Cre control mice. Nonetheless, in both *Atg7^Δ^*^Osx1^ and *Sod2^Δ^*^Osx1^ mice the response to loading was identical to that observed in control mice, indicating that compromised *Atg7*-dependent autophagy or excessive mtROS are not sufficient to impair the bone response to tibial compressive loading. Thus, alternative mechanisms of aging might be responsible for the decreased response of the aged skeleton to mechanical stimuli. These findings also suggest that an intact osteocyte dendrite network is not required for the osteogenic response in this model of bone loading.

## INTRODUCTION

Physical exercise is one of the few lifestyle interventions that delays the effects of aging on many tissues, including bone [1–4]. Mechanical loading induced by exercise, specifically with weight-bearing impact, increases bone mass by stimulating bone formation by osteoblasts, the cells that secrete the bone matrix [5–7]. Throughout life, osteoblasts replace the bone matrix that is resorbed by osteoclasts during bone remodeling. This process occurs at the trabecular and endosteal bone surfaces, which are in contact with the bone marrow [8]. In contrast, bone formation at the periosteal surface occurs independently of bone resorption and is responsible for the enlargement of bones during skeletal growth and aging [9].

Bone remodeling is coordinated by osteocytes, former osteoblasts buried within the bone matrix [10]. During their differentiation from osteoblast, osteocytes develop many cellular projections, which form an intricate network connecting osteocytes among each other and with cells on the bone surface [10]. Mechanical forces cause physical deformation of the bone matrix, triggering mechanical signals detected by osteocytes and other cells on the bone surface [11]. Mechanical stimuli are translated into biochemical signals through mechanosensitive signaling pathways that induce the proliferation and differentiation of osteoblast precursors [11, 12]. Weight-bearing exercise that is beneficial to bone mass in young individuals [13, 14] is less effective with aging [15, 16]. Similarly, the anabolic effects of skeletal loading are greater in young-adult mice than in older animals [17–19] for reasons that remain unknown.

A decrease in osteoblast number and bone formation are major contributors to the loss of bone mass with aging in humans [20, 21] and mice [22]. Mechanisms of aging, such as excessive reactive oxygen species (ROS) or a decline in autophagy, have been implicated in the decreased bone formation in aged bone [23–28]. Mitochondrial (mt) ROS increase with age in bone and their attenuation in mesenchymal lineage cells − including osteoblast precursors, osteoblasts, and osteocytes − counteracts the loss of bone mass in old mice [23, 29, 30]. Mitochondrial ROS produced during mitochondrial respiration are combated by antioxidant defense mechanisms involving superoxide dismutase 2 (*Sod2*) [31]. In line with the contribution of mtROS to the effects of aging in bone, deletion of *Sod2* in osteoblast-lineage cells decreases bone formation and bone mass [32, 33].

Macroautophagy, hereafter referred to as autophagy, maintains cellular function and homeostasis by recycling intracellular components such as misfolded proteins or damaged organelles [34]. The role of autophagy on bone formation has been demonstrated with the deletion of essential autophagy-related genes, such as autophagy-related (*Atg*) 7, *Atg5*, and RB1-inducible coiled-coil 1 (*RB1CC1*) in osteoblast-lineage cells using dentin matrix protein 1 (Dmp1*)*-, Osterix 1 (Osx1*)*-, and collagen type I alpha 1 (Col1a1*)*-Cre-targeted cells [35–39]. *Atg7*, an E1-like enzyme, activates the microtubule-associated protein 1A/1B-light chain 3 (LC3) by conjugating it to phosphatidylethanolamine, facilitating the lipidation of LC3-I into LC3-II. This allows the incorporation of LC3-II into the autophagosome membrane, which is essential for autophagosome formation [40]. Mice lacking *Atg7* in osteoblast-lineage cells have low bone mass and strength due to reduced osteoblast number and bone formation [36]. Autophagy becomes less effective with aging and might contribute to decreased bone formation in old mice [25–28]. Mechanical forces, such as compressive and fluid shear stress, have been shown to enhance autophagic flux in osteocyte and osteoblast cell cultures [41–43].

Despite the evidence supporting the contribution of excessive mtROS or deficient autophagy to the decrease in osteoblastogenesis and bone mass with aging, it remains unknown whether these mechanisms specifically impact the bone mechanoresponsiveness. Here, we examined whether deficient autophagy or elevated mtROS in osteoblast-lineage cells compromises the osteogenic response to mechanical stimulation.

## RESULTS

### Aging reduces the periosteal osteogenic response to mechanical stimulation

**Figure 1:**
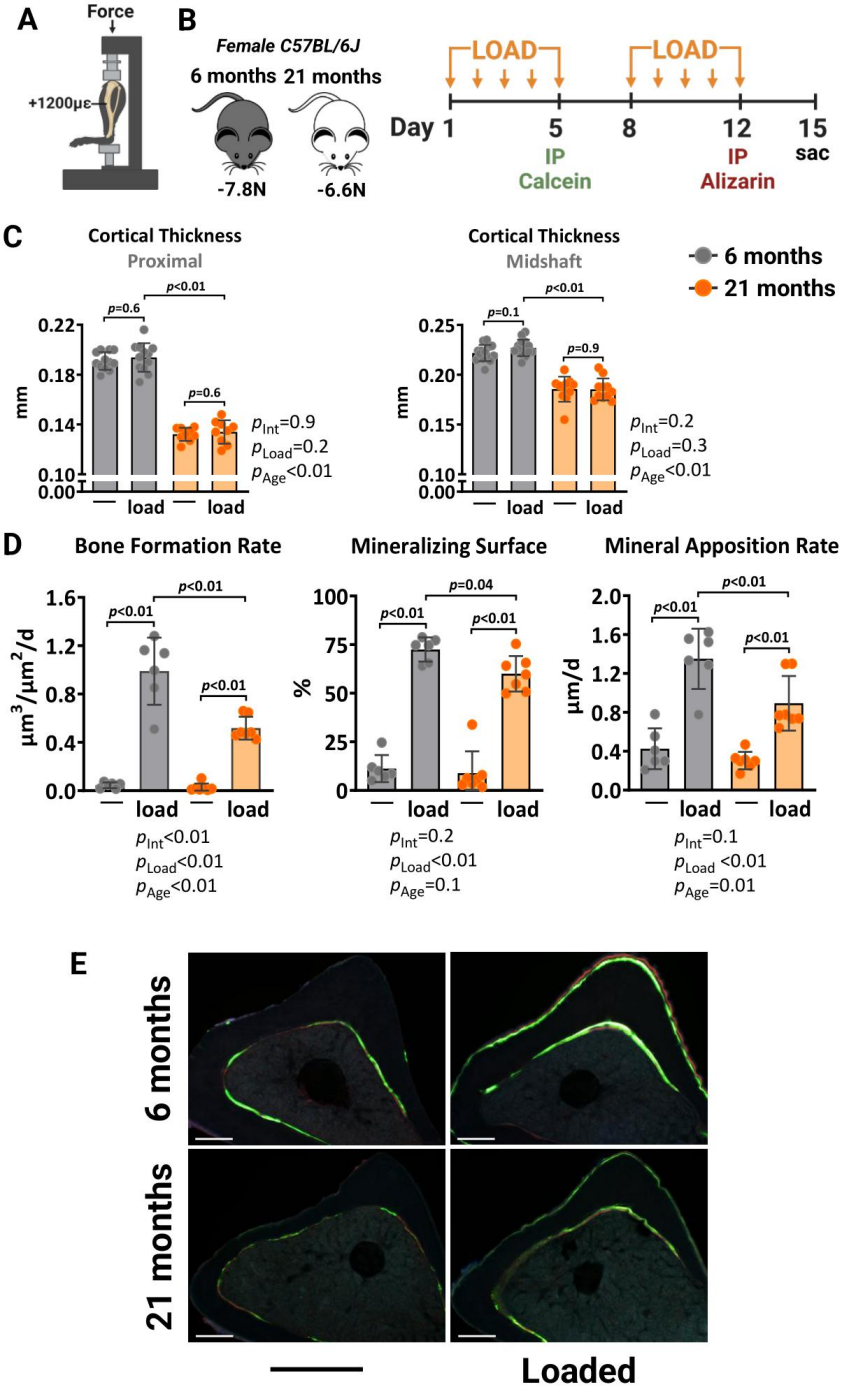
**Aging reduces the periosteal osteogenic response to mechanical stimulation in female mice.** (**A**) Schematic illustration of tibia axial compressive loading applied on mouse tibia with +1200με peak strain at the midshaft. (**B**) Experimental timeline describing the loading and fluorescent dye administration plan. (**C**) Micro-CT measurements at 5 mm proximal to the tibiofibular junction and at the midshaft of the right (non-loaded) and left (loaded) tibia from young (n=12) and old (n=9) female C57BL/6J mice. (**D**) Histomorphometric measurements at the periosteal tibial surface 5 mm proximal from the tibiofibular junction of young (n=6) and old (n=7) female mice, and (**E**) representative photomicrographic cross-section images. Scale bar, 200 μm. Bars represent mean ± SD. Data analyzed using two-way mixed ANOVA; *p*-values adjusted by Holm-Sidak’s test. Int: Interaction.

We first compared the response to mechanical stimulation of young (6 months) and old (21 months) female C57BL/6J mice. The axial tibial compressive loading was used with a peak force (or strain) of +1200με at the tibial midshaft (Figure 1A), previously shown to mimic the strains experienced by bones during normal daily activities [44]. The left tibia of each mouse was loaded at 4 Hertz (Hz) for 1200 cycles/day, 5 days/week for 2 weeks. Young and old mice were loaded with -7.8 and -6.6 Newtons (N), respectively, based on *a priori* strain analysis to achieve strain-matched loading. Mice were loaded on days 1–5 and 8–12, injected with calcein on day 5 and alizarin on day 12 to allow for the quantification of bone formation (Figure 1B). After sacrifice on day 15, both tibiae were collected. The right tibia was used as the non-loaded control. Micro-computed tomography (micro-CT) analysis at midshaft or at 5 mm proximal from the distal tibiofibular junction revealed that loading had no significant impact on cortical thickness in both young and old mice (Figure 1C). Bone formation rate measured by calcein-alizarin double labeling, was increased at the outer (periosteal) loaded tibia surface in 6-month-old mice, due to increases in both mineralizing surface and mineral apposition rate (Figure 1D, 1E). These osteogenic effects were also detected in the loaded tibia of 21-month-old mice. Yet, the magnitude of the loading-induced bone formation rate was significantly lower (*p*_int_ <0.01) in old mice, as described previously by others [17–19].

**Figure 2:**
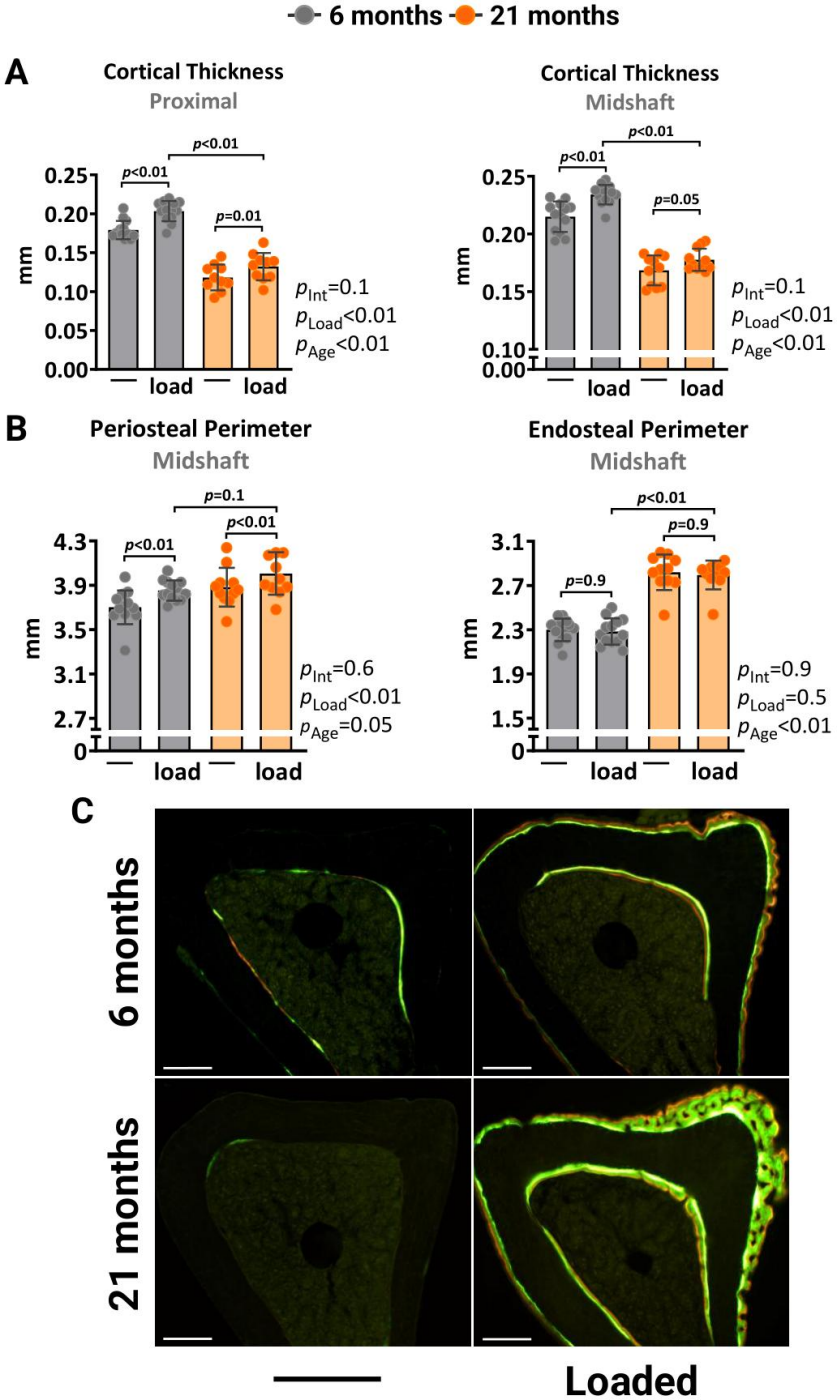
**Higher mechanical strain leads to periosteal woven bone formation in old mice.** Left tibia of young (6 mo) and old (21 mo) C57BL/6J female mice were loaded with -10N and -8.5N, respectively, to achieve +1500με peak strain at the midshaft. (**A**) Micro-CT measurements at 5 mm proximal to the tibiofibular junction and at the midshaft of the right (non-loaded) and left (loaded) tibiae from young (n=13) and old (n=11) female mice. (**B**) Endosteal and periosteal perimeters at the tibia midshaft. (**C**) Representative photomicrographic cross-section images of the proximal tibia shaft. Scale bar, 200 μm. Bars represent mean ± SD. Data analyzed using two-way mixed ANOVA; *p*-values adjusted by Holm-Sidak’s test. Int: Interaction.

We also investigated how higher magnitudes of strain, exceeding what bones experience during normal daily activities, affect the mechanoresponsiveness of aged mice. The left tibia of 6- and 21-month-old female C57BL/6J were loaded with -10N and -8.5N, respectively, to achieve a strain magnitude of +1500με at midshaft. Two weeks of loading increased the cortical thickness at midshaft and proximal sites in the loaded tibia of young mice due to increased periosteal perimeters, while no effects were found at the endosteal surface (Figure 2A, 2B). Loading of old mice caused a similar effect. However, during the micro-CT analysis, we observed that old mice formed woven bone in their loaded tibias. This phenomenon typically occurs in response to bone damage, such as micro-fractures. Histomorphometry analysis confirmed the formation of irregular woven bone, particularly at the periosteal surface (Figure 2C). Collectively, these results indicate that the ability of the aged skeleton to form new bone in response to increased mechanical demands is considerably attenuated compared to the young skeleton.

### Excessive mitochondrial ROS in osteoblast-lineage cells does not alter load-induced bone formation

**Figure 3:**
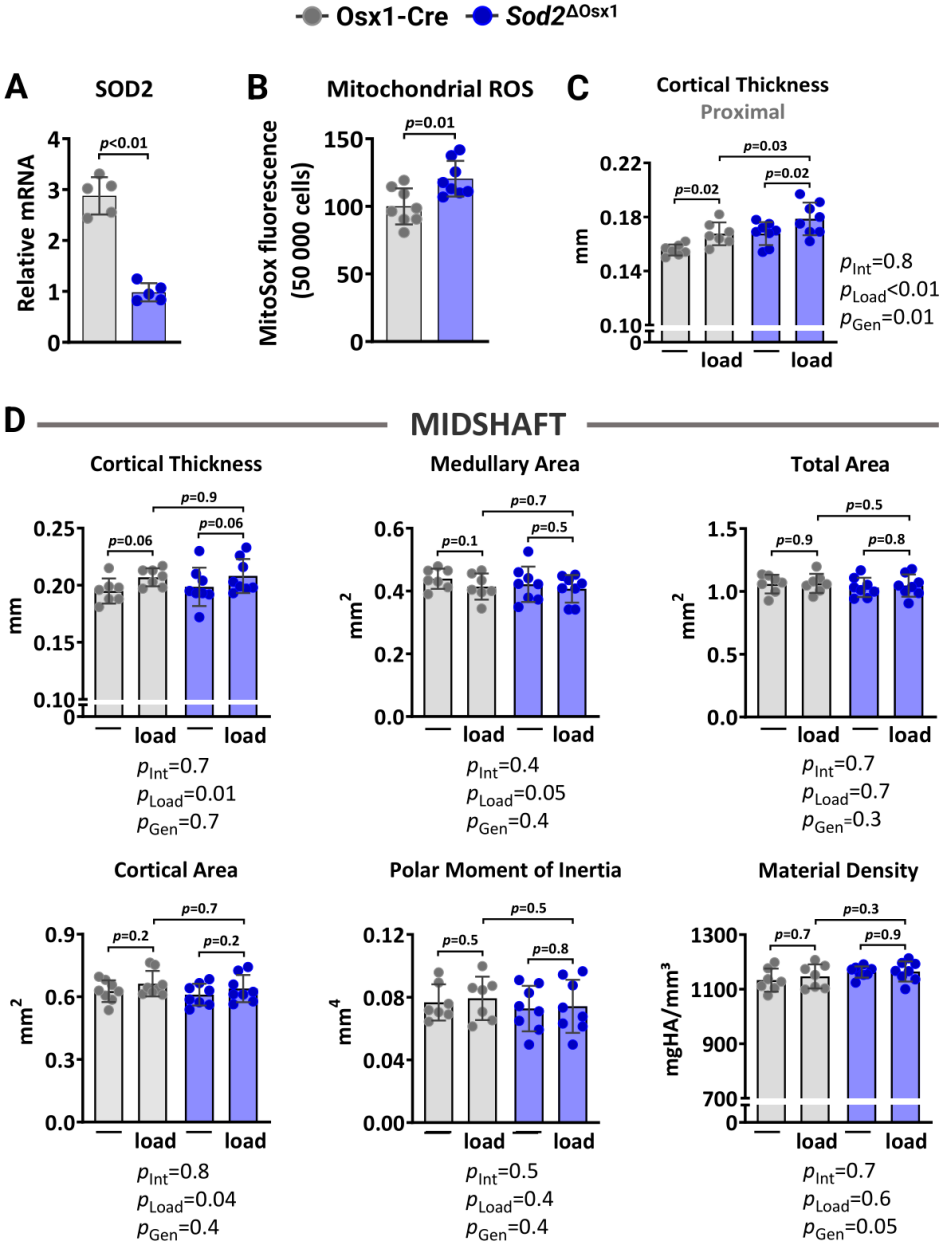
***Sod2* deficiency in osteoblast-lineage cells does not alter load-induced increase in cortical bone mass.** (**A**) mRNA levels measured by quantitative RT-PCR (n=5/genotype) and (**B**) mtROS levels (n=8/genotype) in bone marrow-derived stromal cell cultures from Osx1-Cre and *Sod2*^ΔOsx1^ mice cultured for 7 days. Micro-CT measurements at 5 mm proximal to the tibiofibular junction (**C**) and at the midshaft (**D**) of the right (non-loaded) and left (loaded) tibiae from Osx1-Cre (n=7) and *Sod2*^ΔOsx1^ (n=8) female mice. The tibiae of Osx1-Cre and *Sod2*^ΔOsx1^ were loaded with -8.5N to achieve +1200με peak strain, following the same timeline as in Figure 1B. Bars represent mean ± SD. Data analyzed using two-tailed unpaired t-test or two-way mixed ANOVA; *p*-values adjusted by Holm-Sidak’s test. Int: Interaction; Gen: Genotype.

We next examined whether excessive mtROS caused by *Sod2* deletion could impair the osteogenic response to mechanical loading. *Sod2* metabolizes the highly reactive superoxide anion free radical, a primary byproduct of mitochondrial respiration, into hydrogen peroxide [29]. Here, we generated mice lacking *Sod2* in osteoblast lineage cells, designated *Sod2*^ΔOsx1^, by crossing *Sod2*-flox (*Sod2*^f/f^) mice with transgenic mice expressing the Cre recombinase under the control of Osx1-Cre regulatory elements. The Osx1-Cre transgene causes recombination in stromal cells, osteoblast progenitors, osteoblasts, and osteocytes [45]. Female Osx1-Cre littermates were used as controls. Quantitative RT-PCR analysis confirmed a reduction in *Sod2* mRNA expression levels in bone marrow-derived osteoblastic cell cultures from *Sod2*^ΔOsx1^ mice (Figure 3A). As expected, the reduction in *Sod2* increased mtROS levels as measured by mitochondrial superoxide (MitoSOX) red staining (Figure 3B).

**Figure 4:**
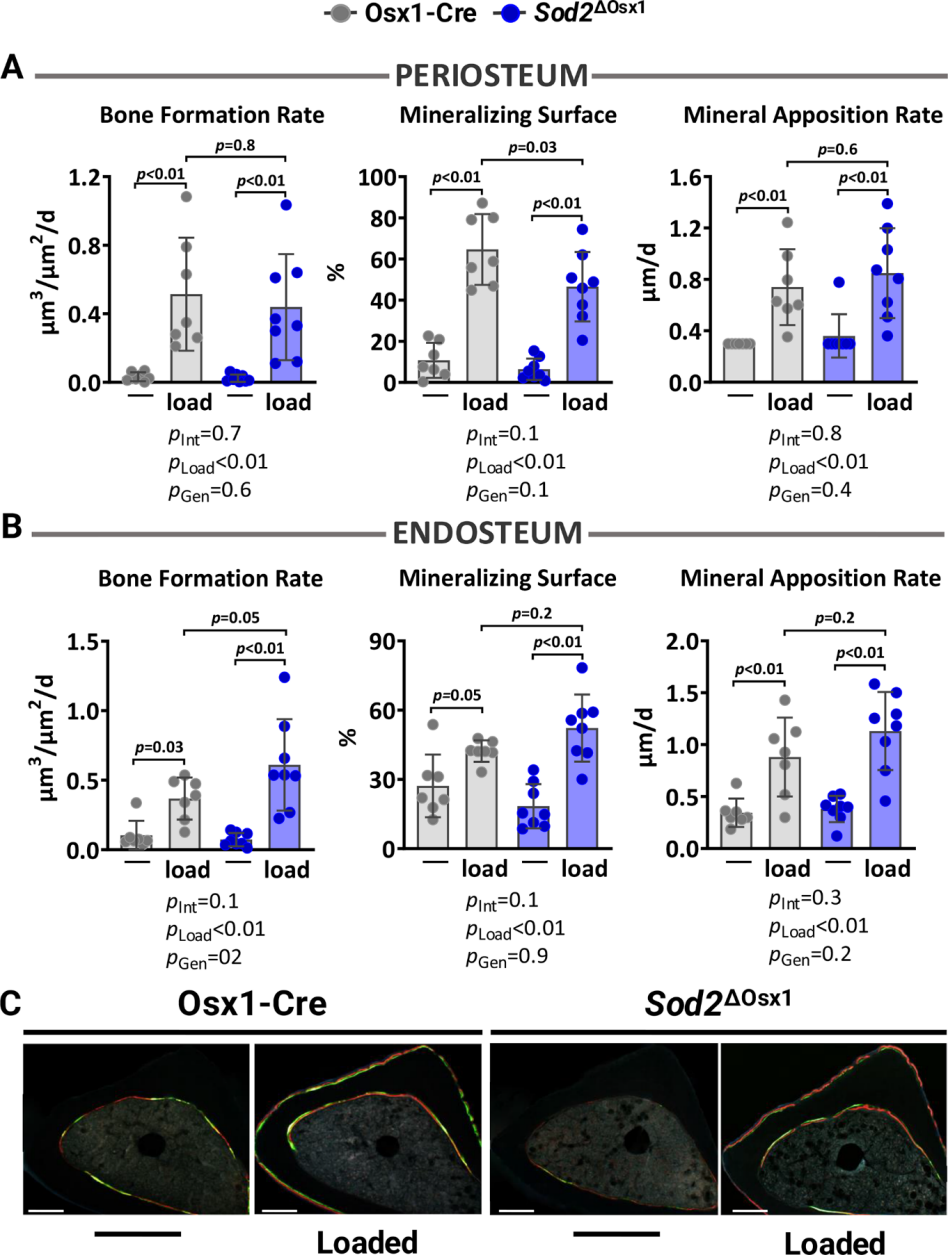
**Excessive mitochondrial ROS in osteoblast lineage does not impact load-induced bone formation.** Histomorphometric measurements at the periosteal (**A**) and endosteal (**B**) tibial surface at 5 mm proximal from the tibiofibular junction of the right (non-loaded) and left (loaded) tibia from Osx1-Cre (n=7) and *Sod2*^ΔOsx1^ (n=8) female mice, and (**C**) representative photomicrographic cross-section images. Scale bar, 200 μm. Bars represent mean ± SD. Data analyzed using two-way mixed ANOVA; *p*-values adjusted by Holm-Sidak’s test. Int: Interaction; Gen: Genotype.

We then loaded the left tibiae of 12-month-old female *Sod2*^ΔOsx1^ mice and their Osx1-Cre littermates with -8.5N at +1200με for two weeks, as described above (Figure 1B). Loading increased cortical thickness at proximal tibia and midshaft similarly in *Sod2*^Δ^*^Osx1^* and Osx1-Cre mice (Figure 3C, 3D). Consistently, no genotype-associated differences were observed in medullary area, total area, cortical area, polar moment of inertia, or material density at the midshaft of the loaded tibia (Figure 3D). In line with these results, the increased bone formation rate, mineralizing surface, and mineral apposition rate at the periosteal and endosteal surfaces of the loaded tibia were indistinguishable between *Sod2*^ΔOsx1^ and Osx1-Cre mice (Figure 4A–4C). Taken together, these results indicate that an increase in mtROS in osteoblast-lineage cells was not sufficient to impact the bone anabolic response to mechanical stimulation.

### Loading induces bone formation independently of *Atg7*-dependent autophagy

**Figure 5:**
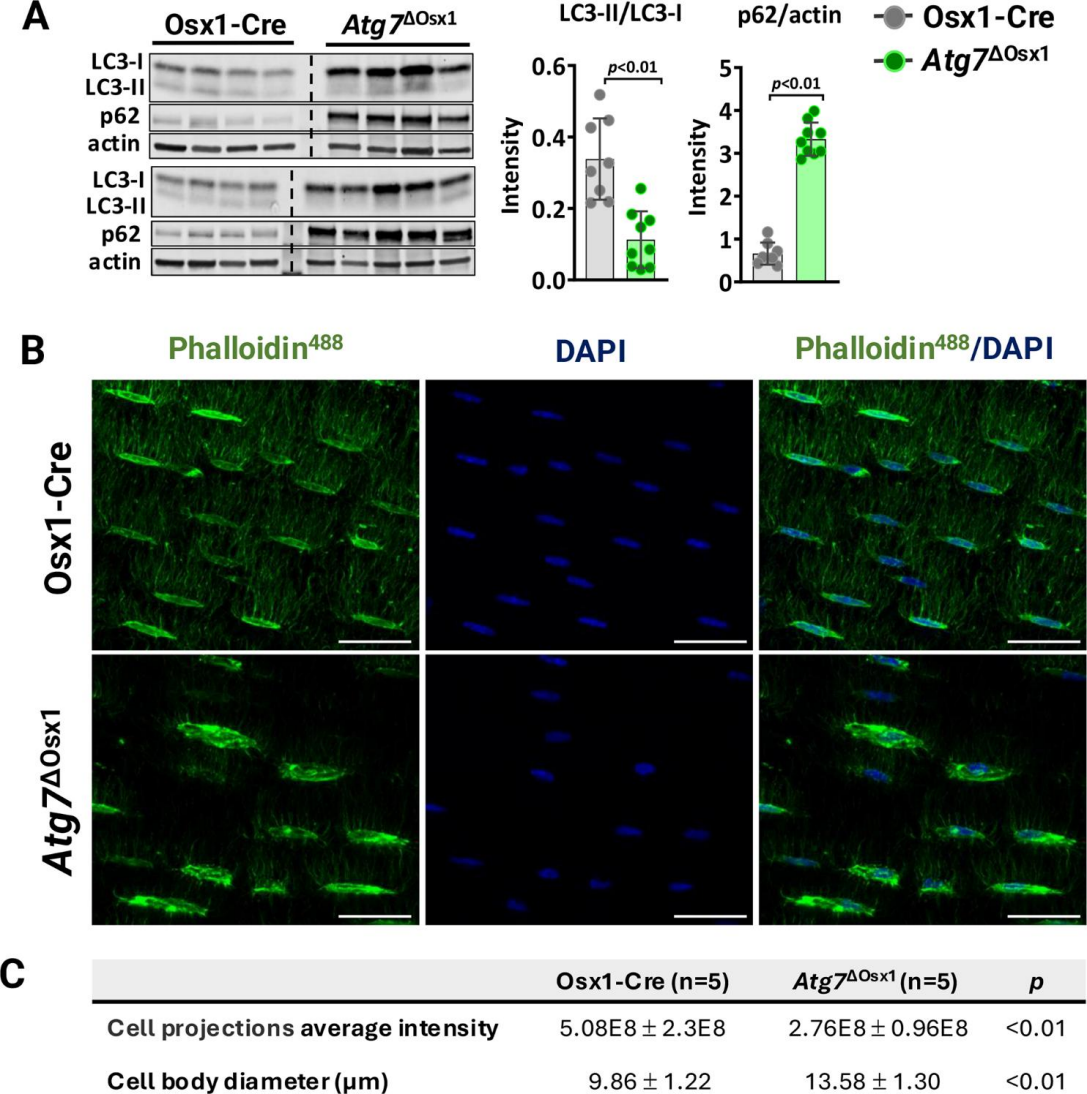
***Atg7* deficiency in osteoblast-lineage cells disrupts the osteocyte network.** (**A**) Western blot (left) and quantification of band intensity (right) in protein lysates extracted from humeri shafts of Osx1-Cre (n=8) and *Atg7*^ΔOsx1^ (n=9) female mice. (**B**) Representative images of osteocyte network in femoral cortical diaphysis stained with phalloidin-Alexa488 [green] and DAPI [blue]. Scale bar, 200 μm. (**C**) Average fluorescence intensity of osteocyte projections and cell body diameter. Bars represent mean ± SD. Data analyzed by two-tailed unpaired t-test.

To examine whether autophagy deficiency in osteoblasts could potentially account for the decreased load-induced bone formation with aging, we used mice harboring a conditional allele for *Atg7* in cells of the osteoblast lineage, designated *Atg7*^ΔOsx1^ [36]. To confirm the suppression of autophagy, we measured the levels of LC3 and p62 in osteoblastic cell cultures using immunoblot analysis (Figure 5A). During autophagosome formation, the cytoplasmic form of LC3 (LC3-I) is lipidated and incorporated into the forming autophagosome membrane as LC3-II [40]. Based on this, LC3-I to LC3-II conversion is commonly used as an indicator of autophagy levels [46]. p62, a classical autophagy substrate, brings cargo to the autophagosome for degradation and accumulates when autophagy is suppressed [46]. Consistent with previous reports [36], deletion of *Atg7* from Osx1-Cre-expressing cells inhibited autophagosome formation and thereby autophagy, as indicated by decreased LC3 conversion and p62 accumulation (Figure 5A).

We have previously shown that deletion of *Atg7* drastically reduces dendrite connectivity in cortical bone [36]. It has been postulated that degeneration of the osteocyte network impairs the bone anabolic responses to loading [47, 48]. Here, we confirmed the effects of *Atg7* deletion on the osteocyte dendritic network. We first assessed the density of osteocyte cell projections, which form as osteocytes become embedded into the bone matrix (Figure 5B). The average fluorescence intensity normalized to the bone area, indicative of the number and size of osteocyte cellular projections, was significantly lower in the *Atg7*^ΔOsx1^ mice compared to Osx1-Cre controls (Figure 5C). These findings confirm that deletion of *Atg7* in osteoblast lineage disrupts the formation or maintenance of the osteocyte network, as described previously [36]. In addition, osteocyte cell bodies were larger in *Atg7*^ΔOsx1^ mice than in the controls (Figure 5C). This is likely due to the accumulation of cytoplasmic components that are normally degraded during the transition from osteoblasts to osteocytes [32].

**Figure 6:**
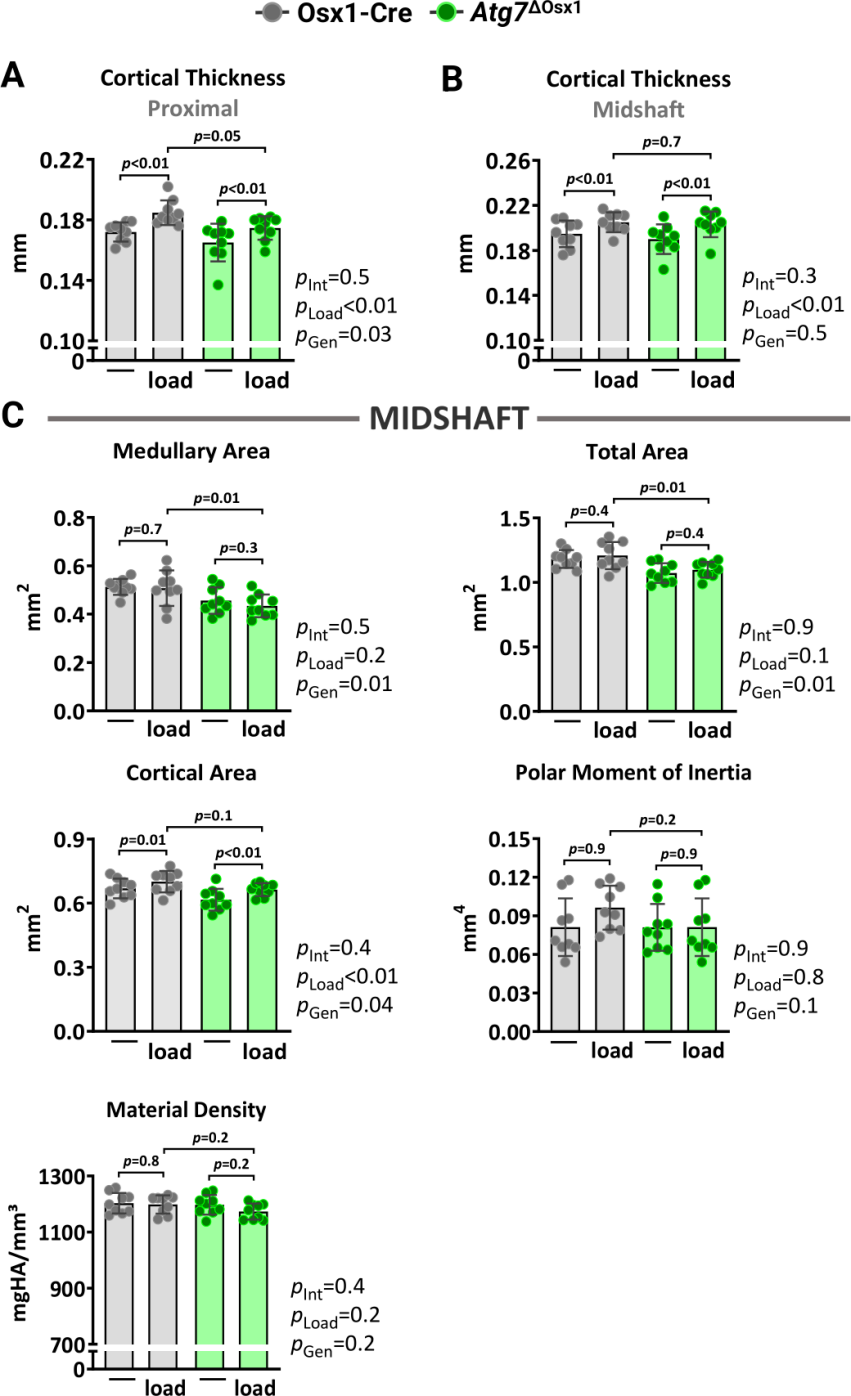
**Lack of autophagy in osteoblast lineage does not alter load-induced increase in cortical bone mass.** Micro-CT measurements at 5 mm proximal to the tibiofibular junction (**A**) and at the midshaft (**B**, **C**) of the right (non-loaded) and left (loaded) tibiae from Osx1-Cre (n=9) and *Atg7*^ΔOsx1^ (n=9) female mice. The tibiae of Osx1-Cre and *Atg7*^ΔOsx1^ mice were loaded with -7.6N and -7.3N, respectively, to achieve +1200με peak strain, following the timeline shown in Figure 1B. Bars represent mean ± SD. Data analyzed by two-way mixed ANOVA; *p*-values adjusted by Holm-Sidak’s test. Int: Interaction; Gen: Genotype.

**Figure 7:**
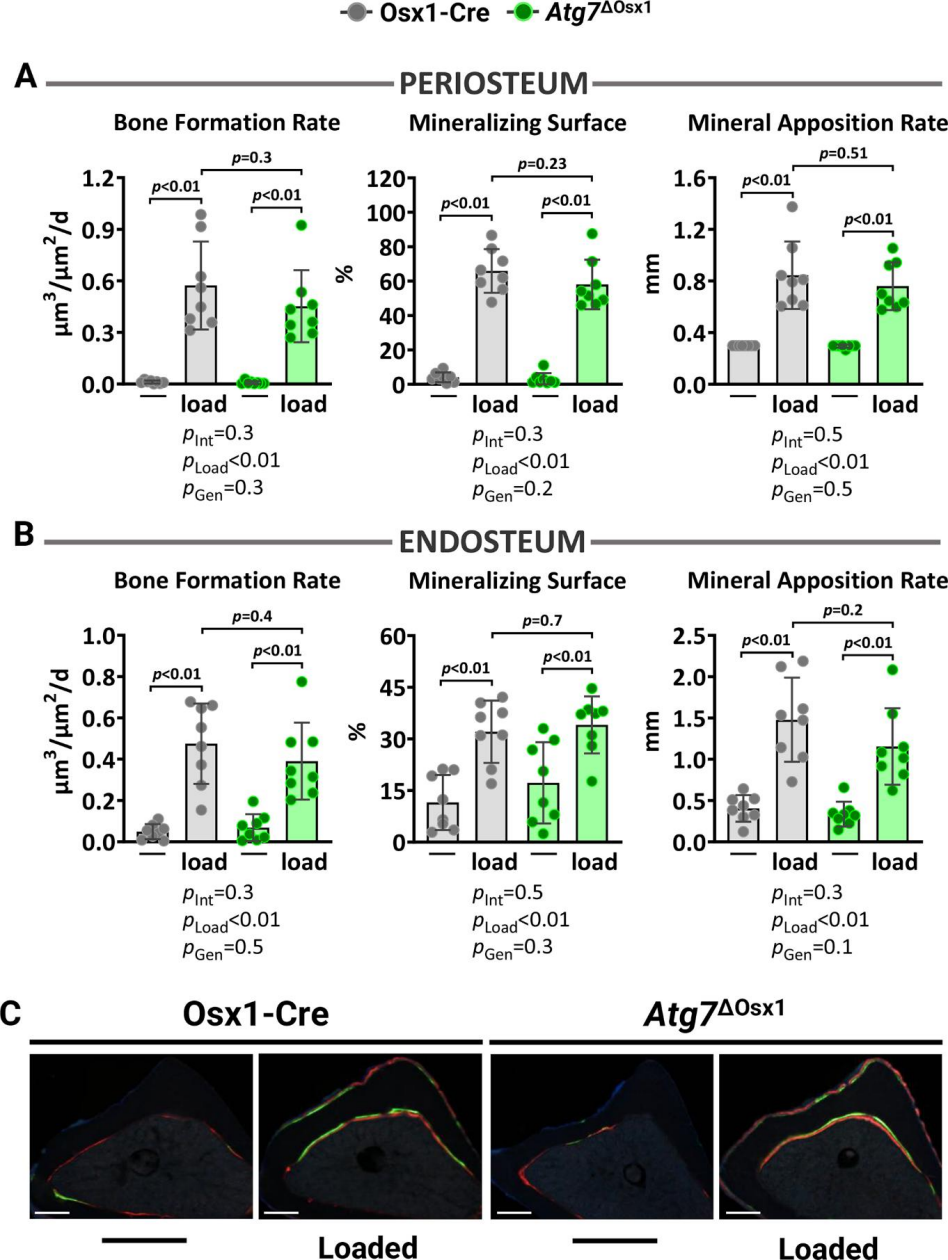
**Lack of autophagy in osteoblast lineage does not impact loading-induced bone formation.** Histomorphometric measurements at the periosteal (**A**) and endosteal (**B**) tibial surface at 5 mm proximal from the tibiofibular junction of the right (non-loaded) and left (loaded) tibia from Osx1-Cre (n=7) and *Atg7*^ΔOsx1^ (n=8) female mice, and (**C**) representative photomicrographic cross-section images. Scale bar, 200 μm. Bars represent mean ± SD. Data analyzed by two-way mixed ANOVA; *p*-values adjusted by Holm-Sidak’s test. Int: Interaction; Gen: Genotype.

We loaded the left tibiae of 10-month-old female *Atg7*^ΔOsx1^ mice and Osx1-Cre littermates with −7.3 and −7.6N for 1200 cycles, respectively. Two weeks of loading increased cortical thickness at midshaft and proximal tibia similarly in *Atg7*^ΔOsx1^ mice and Osx1-Cre littermates (Figure 6A). Accordingly, no differences between genotypes were detected in medullary area, total area, polar moment of inertia, or material density at the midshaft of the loaded tibia (Figure 6B). In addition, loading increased bone formation rate, mineralizing surface, and mineral apposition rate at the periosteal and endosteal (Figure 7A–7C) surfaces to a similar extent in *Atg7*^ΔOsx1^ and Osx1-Cre mice. These results indicate that loss of autophagy in osteoblast-lineage cells was not sufficient to alter the osteogenic response to mechanical stimulation.

## DISCUSSION

Mechanical loading caused by exercise is a potent inducer of bone formation [5–7]. Nonetheless, the mechanical stimuli that increase osteogenesis in young adults are not as effective in older individuals [13–16], suggesting age-related changes in the ability to sense and/or transduce mechanical stimuli. Here, we found a significant decrease in cortical bone formation following two weeks of axial tibia loading in old C57BL/6J female mice compared to young-adult animals. Our results are in line with previous studies reporting reduced or negligible responses to skeletal loading with aging in old mice [17–19]. Previous work has suggested that a decline in Piezo-type mechanosensitive ion channel component 1 (*Piezo1)* contributes to skeletal aging [49]. Piezo1 is essential for the bone formation in response to mechanical loading [50]. However, in old mice, Piezo1 antagonizes bone resorption and does not appear to contribute to bone formation under physiological conditions [12]. Diminished cell proliferation and wingless-related integration site (Wnt) signaling have also been postulated as contributors to the lower anabolic effects of mechanical stimuli in aged bone [18, 51]. Nonetheless, the contribution of common mechanisms of aging to the impaired mechanoresponsiveness of the skeleton has not been examined.

Oxidative stress, caused by an imbalance between the production and clearance of ROS, is associated with skeletal aging in both humans and mice [23, 52, 53]. ROS are primarily produced in the mitochondria during cellular respiration and are cleared by distinct antioxidant mechanisms in healthy cells [54]. We have previously used a mitochondria-targeted catalase transgene (mitoCAT) in osteoblast-lineage cells to show that attenuation of mtROS protects mice against skeletal aging [29]. Accordingly, excessive mtROS caused by *Sod2* deletion decreases bone mass in young mice due to reduced bone formation [32, 33]. Herein, we found that excessive mtROS does not alter load-induced bone formation. Others have shown that attenuation of mtROS does not alter the loss of bone mass due to unloading [55]. Together with our work, these findings indicate that mtROS in osteoblastic cells is not a major mediator of the effects of load on the skeleton.

Autophagy in muscle has been implicated both as a stress-responsive process and a mediator of exercise-induced mechanical stimulation [56, 57]. Indeed, acute endurance exercise induces autophagic flux in skeletal muscle in mice [58–63] and humans [64–66]; and mice with compromised autophagy in muscle have reduced running endurance capacity [58, 59]. Mechanical stimulation of osteoblastic cells in culture activates autophagy which, in turn, promotes osteoblastogenesis [41–43]. These *in vitro* findings, along with evidence that autophagy in osteoblast-lineage cells contributes to bone formation, support the idea that autophagy is involved in the skeletal response to mechanical loading. Nonetheless, thus far, no work has been performed to directly address this hypothesis. Contrary to the evidence from *in vitro* studies, we show herein that loss of *Atg7*-dependent autophagy in the osteoblast-lineage does not impact the response to mechanical stimulation.

Mechanical forces cause physical deformation of the bone matrix and, consequently, promote stimuli such as fluid flow and fluid shear stress. Different cells act as mechanosensors to detect external mechanical signals [10]. Osteocytes have been proposed to act as mechanosensors due to their location within the bone matrix. These cells comprise over 90% of all bone cells and form a network of highly interconnected cell projections, reminiscent of dendrites, known as the lacuna-canalicular system. These projections allow for direct cell-to-cell communication among osteocytes and with cells on the bone surface, and facilitate the release of soluble factors to modulate both bone resorption and formation [67, 68]. Thus, changes in mechanical strain alter fluid flow inside the lacuna-canalicular system, generate fluid shear stress, and thereby stimulate osteocytes [67, 68]. Osteocyte projections decrease with aging in humans [69] and mice [47, 48, 70]. We have previously shown, and confirmed here, that the maintenance of osteocyte projections requires *Atg7*-dependent autophagy [36]. Although we have not examined osteocytes in our *Sod2*^ΔOsx1^ model, previous work has shown that mice with *Sod2* deletion in cells of the osteoblast lineage exhibit disorganized osteocytic canalicular networks [32]. It has been proposed that the age-related degeneration of the osteocyte network impairs the bone anabolic responses to loading [47, 48]. The present findings that the osteogenic effects of loading are unaltered in *Sod2*^ΔOsx1^ and *Atg7*^ΔOsx1^ mice do not support the idea that an intact osteocyte network is required for the bone response to the loading model used in our experiments.

This study has some limitations. First, we cannot exclude the possibility that forms of autophagy other than macroautophagy, such as chaperone-mediated autophagy (CMA) or microautophagy, contribute to load-induced bone formation. CMA [71, 72] and microautophagy [73] have been described in cultured osteoblast lineage cells. CMA contributes to vertebral bone mass accrual during growth, but not to the loss of bone mass with aging [71, 72]. The functional role of microautophagy remains unexplored. Second, measurements of autophagy and ROS in osteocytes are not provided in this study. Nevertheless, the efficacy of the genetic models used here to suppress autophagy by deleting *Atg7* and to increase mtROS by deleting *Sod2* in targeted cells, including osteocytes, has been previously described by us and others [32, 33, 35, 36]. We have used electron microscopy to show that *Atg7* deletion in osteoblast-lineage cells prevents autophagosome formation in osteocytes [35], and elucidated that autophagy is critical for the reduction in endoplasmic reticulum and mitochondria content during the differentiation of osteoblasts into osteocytes [36]. In addition, osteocyte-enriched cell preparations from mice with *Sod2* deletion have increased ROS [32, 33]. Third, while our micro-CT data suggest that the material density did not change with loading alone, *Sod2* or *Atg7* deletion, the sensitivity of this measurement is limited when compared to Raman spectroscopy, Fourier transform infrared spectroscopy, or nanoindentation. Previous studies indicate that loading alters bone mineral and matrix properties [74, 75]. Thus, it remains possible that *Sod2* or *Atg7* deletion in osteoblast-lineage cells alters material properties but not bone formation. Fourth, our findings here rely on a single model of mechanical loading. Future studies should utilize other models, such as exercise, to strengthen these observations.

In conclusion, our work suggests that increased mtROS or decreased *Atg7*-dependent autophagy in osteoblastic cells, as well as reduced osteocyte dendrite number – common features of aged bone – do not contribute to reduced mechanoresponsiveness with aging. In muscle, altered mitochondrial metabolism associated with low nicotinamide adenine dinucleotide (NAD^+^) levels has been implicated in the reduced response of older adults to physical activity [76]. Administration of NAD^+^ precursors to aging mice attenuates the loss of bone mass [77, 78]. Whether stimulation of NAD^+^ could also promote bone mechanoresponsiveness requires further work.

## MATERIALS AND METHODS

### Animal experimentation

Mice with conditional deletion of *Sod2* in osteoblast-lineage cells were generated by a two-step breeding strategy. *Sod2* floxed *(f/f)* mice [79] were recovered from cryopreserved sperm at the Genetic Models Core Facility at University of Arkansas for Medical Sciences (UAMS). Please note that during genotyping of these mice, we determined that the neomycin phosphotransferase (neo) selection cassette that was contained in the original conditional allele was missing, likely due to an unintended recombination event. Sequencing of PCR products produced using primers P1 5’-CGA GGG GCA TCT AGT GGA GAA G-3’ and P2 5’-TTA GGG CTC AGG TTT GTC CAT AA-3’ (product sizes = 500 bp for WT and 540 bp for the floxed allele) revealed that the 540 bp product contained the loxP site predicted to exist after recombination of the loxP sites flanking the neo cassette [80]. Nonetheless exon 3 of the *Sod2* gene remained flanked by functional loxP sites allowing us to use this version as a conditional (floxed) allele. Offspring from the *in vitro* fertilization were backcrossed to C57BL/6J mice (CD45.2) for four generations. Homozygous Osx1-Cre transgenic mice (B6.Cg-Tg (Sp7-tTA, tetO-EGFP/Cre) 1Amc/J; Strain #: 6361 Jackson Laboratory) were crossed with *Sod2*^f/f^ mice to generate mice heterozygous for the *Sod2* floxed allele with (*Sod2*^f/+^; Osx1-Cre) or without (*Sod2*^f/+^) Osx1-Cre allele. These offspring were intercrossed to generate *Sod2*^Δ^*^Osx1^* mice and Osx1-Cre controls. Offspring were genotyped by PCR using the following primer sequences: *Sod2*-flox P1 5’-CGA GGG GCA TCT AGT GGA GAA G-3’, P2 5’-TTA GGG CTC AGG TTT GTC CAT AA-3’, Cre-fwd 5’-GCG GTC TGG CAG TAA AAA CTA TC-3’ and Cre-rev 5’-GTG AAA CAG CAT TGC TGT CAC TT-3’. Mice lacking *Atg7* in Osx1-Cre targeted cells were generated using a similar strategy, as previously described [36]. PCR-based genotyping was conducted using the following primer sequences: *Atg7*-flox P1 5’-TGG CTG CTA CTT CTG CAA TGA TGT-3’, P2 5’-TCT CCC AAG ACA AGA CAG GGT GAA-3’, P3 5’-CAG GAC AGA GAC CAT CAG CTC CAC-3’; Cre-fwd 5’-GAG AAT AGG AAC TTC GGA ATA GTA AC-3’ and Cre-rev 5’-CCC TGG AAG TGA CTA GCA TTG-3’; IntCon-fwd 5’-AGA GAG CTC CCC TCA ATT ATG T-3’ and IntCon-rev 5’-AGC CAC TTC TAG CAC AAA GAA CT-3’. Offspring from all genotypes were tail-clipped for DNA extraction at weaning (21 days) and then group-housed with same-sex littermates. Mouse genotyping was performed using REDExtract-N-Amp™ Tissue PCR Kit (MilliporeSigma, XNAT-100RXN) according to the manufacturer’s instructions. Briefly, genomic DNA was extracted from fresh tail clippings by incubation in an extraction buffer (10 min, room temperature), followed by heating (95° C, 3 min), and neutralization. Bands were visualized using a ChemiDoc XRS^+^ System (Bio-Rad, Hercules, CA, USA). All mice were housed in groups of 2-5 animals per cage under standard laboratory conditions with a 12-hour light/dark cycle, a constant temperature of 23°C, and humidity of 48%. Mice had *ad libitum* access to water and a standard rodent diet (Envigo, Teklad 22/5) containing 22% protein, 1.13% calcium, and 0.94% phosphorus. To avoid the bone phenotype associated with the Osx1-Cre transgene, *Atg7*^ΔOsx1^ mice were maintained on a doxycycline diet until 3 weeks of age to prevent Cre activity. *Sod2*^ΔOsx1^ and *Atg7*^ΔOsx1^ mice were used between the ages of 10-12 months old to avoid both the confounding effects of skeletal growth and those caused by natural aging. Mice were euthanized by CO_2_ inhalation at designated time points followed by cervical dislocation. All animal procedures (IPROTO202400000001 and IPROTO202100000238) were approved by the UAMS Institutional Animal Care and Use Committee.

### Tibia axial compressive loading

An axial compressive load with a +1200 με peak strain was applied to the left tibia midshaft using an ElectroForce 5500 (TA Instruments, New Castle, DE, USA). Briefly, with anesthetized mice (2.5% isoflurane) in a prone position, the left tibiae were positioned vertically in the load testing system, with the superiorly positioned knee attached to the system’s actuator and the foot held in a static fixture inferiorly. The load was applied for 1200 cycles/day (4 Hz triangle waveform with a 0.1-second rest time between each cycle) for five consecutive days per week over two weeks, following a protocol previously shown to be anabolic [81]. The right leg served as a non-loaded contralateral control. After each loading session, mice were returned to their cages and allowed unrestricted activity. To label actively mineralizing surfaces, calcein (20 mg/kg body weight; Sigma-Aldrich) and alizarin red (20 mg/kg body weight; Sigma-Aldrich) were injected intraperitoneally 10 days and 3 days before sacrifice, respectively. Mice were euthanized on day 15, and tissues were collected for skeletal analyses.

### Strain gauge measurement

To determine the load required to achieve a peak strain of +1200με in each experimental group of mice, axial compressive loading was applied to harvested tibiae *ex vivo*. A single-element strain gauge (C2A-06-015LW-120, VPG Micro-Measurements, Wendell, NC, USA) was attached to the anteromedial surface of the tibia, located 5 mm proximal to the distal tibiofibular junction, using the M-Bond 200 Adhesive Kit (VPG Micro-Measurements). Force-strain regressions were recorded using ElectroForce 5500 software. The same load was subsequently applied to mice *in vivo*, adjusted according to their age or genotype.

### Skeletal analysis

Tibias were cleaned of soft tissue, fixed in 10% Millonig’s formalin (Leica Microsystems, Deerfield, IL, USA ) with 5% sucrose for 24 hours, and gradually dehydrated into 100% ethanol. Bones were loaded into a 12.3 mm diameter scanning tube, and medium-resolution scans were obtained (12 μm isotropic voxel size) by micro-computed tomography (micro-CT40, Scanco Medical, Wayne, PA, USA). Scans were integrated into 3D voxel images (1024 × 1024 pixel matrices for each planar stack), and a Gaussian filter (σ = 0.8, support = 1) was applied to reduce signal noise. Scan settings included X-ray tube potential (55 kVp), X-ray intensity (145 μA), and integration time (200 ms). Scanco Eval Program v.6.0 was used to measure bone volume. For tibial cortical thickness, 18 slices were analyzed either 5 mm proximal to the distal tibiofibular junction or at the tibia midshaft. A threshold of 260 mg/cm^3^ was applied for cortical analyses. Calibration and quality control were performed weekly using five density standards, and spatial resolution was verified monthly using a tungsten wire rod. Beam-hardening correction was based on the calibration records. Corrections for 200 mg hydroxyapatite were applied to all energy levels. Nomenclature conforms to the recommendations of the American Society for Bone and Mineral Research [82].

### Histomorphometry

After micro-CT analysis, the tibia was embedded undecalcified in methyl methacrylate, and 80-μm-thick longitudinal sections were obtained at 5 mm proximal to the distal tibiofibular junction. Sections were left unstained to assess fluorescent calcein and alizarin red labeling. Quantitative histomorphometry was performed on periosteal and endocortical surfaces using the OsteoMeasure Analysis System (OsteoMetrics, Inc., Atlanta, GA, USA) interfaced with an Axio image M2 (Carl Zeiss, White Plains, NY, USA). The following primary measurements were made: bone surface (BS), single-label surface (sL.S), double-label surface (dL.S), inter-label thickness (Ir.L.Th), and inter-label time (Ir.L.t). The following derived indices were calculated: mineralizing surface (MS, %) [(dLS + sLS/2)/BS], mineral apposition rate (MAR, μm/d) [Ir.L.Th/Ir.L.t], and bone formation rate (BFR, μm^3^/μm^2^/d) [MAR*(MS/BS)]. When MAR was equal to zero an arbitrary value of 0.3 mm was attributed. During dynamic histomorphometric analysis, we observed that two *Atg7*^ΔOsx1^ and two Osx1-Cre mice were missing one of the injections since no double labels were observed. These mice were excluded from the analysis. All histology measurements were made in a blinded fashion. The results are reported using the terminology recommended by the Histomorphometry Nomenclature Committee of the American Society for Bone and Mineral Research [82].

### Osteocyte network analysis and immunofluorescence

Phalloidin staining of actin was performed on the femurs. First, femurs were fixed in buffered 10% formalin for 24 hours, decalcified in 14% EDTA pH 7.1 for one week, stored in 30% sucrose solution, and embedded in Cryo-Gel™ (Electron Microscopy Sciences, Hatfield, PA, USA) for frozen sectioning. Frozen sections, 20 μm thick, were cut and rinsed 3 times in PBS for 10 minutes. Phalloidin staining of the osteocytes dendrites was performed on the femurs as previously described [36]. Briefly, sections were incubated in 0.2% Trition-X100 for 20 minutes with agitation, followed by washing in PBS. Sections were then incubated in 2% BSA for 30 minutes and incubated with Alexa Fluor™ 488-phalloidin (Invitrogen, Waltham, MA, USA) in 0.5% BSA for 2 hours at 4° C. At the end of the incubation, the sections were rinsed with PBS and cover-slipped with Vectashield® Mounting Medium containing DAPI (Invitrogen). Images of phalloidin-stained bone sections were acquired using a Zeiss LSM 510 Meta/AxioVert 200 confocal microscope using a 40 × or 63 × oil objective and z-stacks were obtained using ZEN 2009 software. Measurements of osteocyte dendrites and osteocyte cell body diameter were performed on flattened z-stacks (10 μm for femur) using ImageJ software. To quantify osteocyte dendrites, first, a region of interest (ROI) containing only cortical bone was drawn. Then osteocyte cell bodies were excluded from the ROI. Finally, the total dendrite fluorescence inside the ROI was calculated by subtracting the background fluorescence in a region lacking osteocytes or dendrites from the integrated intensity of the entire region. This value was then divided by the area of the region of ROI. Three fields of cortical bone from each sample were quantified and 3–4 samples per genotype were used.

### cell culture

Bone marrow-derived mesenchymal stem cells (BMSCs) were harvested from the long bones of 5-6 pooled mice per group. Briefly, bone marrow stromal cells were flushed from the tibiae and femora, depleted of red blood cells with ammonium-chloride-potassium (ACK) buffer (0.01 mM EDTA, 0.011 M KHCO3, and 0.155 M NH4Cl, pH 7.3) and filtered through a 70 μM cell strainer. The isolated cells were then cultured for 7 days in 100 mm dishes containing osteogenic medium (α-MEM supplemented with 20% fetal bovine serum (FBS), 1% penicillin/streptomycin (PSG), and 50 μg/ml ascorbic acid) with a half-medium change on day 4. Adherent stromal cells were then trypsinized using Trypsin-EDTA solution 0.25% (Sigma-Aldrich) and used for subsequent analyses. To measure mitochondrial reactive oxygen species, 2×10^4^ cells were re-plated in a black-walled 96-well plate and cultured for two days. To perform qPCR assays, adherent BMSCs were plated in triplicate in 12-well plates at 2×10^5^ cells per well in α-MEM supplemented with 10% FBS, 1% PSG, and 50 μg/ml ascorbic acid. Upon reaching confluency, cells were cultured for an additional 7 days with 10 mM β-glycerophosphate, and media changes every 3 days to induce osteogenic differentiation.

### Mitochondrial ROS

Mitochondrial superoxide (O_2_*•⁻*) levels were measured using the mitochondrial-specific probe MitoSOX™ Red (Thermo Fisher Scientific, M36008). Briefly, cells were washed twice with phosphate-buffered saline (PBS) and incubated with 2.5 μM MitoSOX™ Red for 20 minutes at 37° C. Following two additional PBS washes, fluorescence (Ex/Em = 510/580 nm) was measured using a Cytation™ 5 microplate reader (BioTek) to assess MitoSOX oxidation, indicative of superoxide levels. Nuclei were counterstained with Hoechst dye (Thermo Fisher Scientific, H3570), and MitoSOX fluorescence was normalized to nuclear fluorescence intensity.

### Quantitative RT-PCR

Total RNA was purified from cultured BMSCs using TRIzol™ Reagent (Thermo Fisher Scientific, Waltham, MA, USA). After extraction, RNA was quantified using a NanoDrop™ instrument (Thermo Fisher Scientific), and 1 μg of RNA was then used to synthesize cDNA with the High-Capacity cDNA Archive Kit (Applied Biosystems) according to the manufacturer’s instructions. Transcript abundance in the cDNA was quantified by quantitative PCR (qPCR) using TaqMan™ Universal PCR Master Mix (Thermo Fisher Scientific). The primers and probe for *Sod2* (Mm00449726_m1) were obtained from the TaqMan™ Gene Expression Assays service (Applied Biosystems, Waltham, MA, USA). Relative mRNA expression levels were normalized to the housekeeping gene *mitochondrial ribosomal protein S2* (Mm00475528_m1) using the ΔCt method [83].

### Immunoblot analysis

Protein was extracted from humeri shafts, which were snap-frozen in liquid nitrogen and stored at −80° C. After pulverization, the resulting bone powder was suspended in RIPA buffer (Thermo Fisher Scientific, PI89901) containing protease and phosphatase inhibitors (Cell Signaling Technology, 5872S), following the manufacturer’s instructions. Proteins were separated on 4–20% or 4–15% Mini-PROTEAN TGX™ gels (Bio-Rad, 4561093 and 4561083, respectively) and transferred onto Trans-Blot Turbo™ midi-size nitrocellulose membranes (0.2 μm pore size, Bio-Rad, 1704271). The membranes were blocked for 30 minutes with LI-COR Blocking Buffer-PBS (LI-COR, 927-70001) and incubated overnight at 4° C with primary antibodies on a rocking platform. The primary antibodies used were anti-LC3 (Cell Signaling Technology, 12741T; 1:1000 dilution), anti-p62 (Cell Signaling Technology, 23214S; 1:1000 dilution), and β-actin (MilliporeSigma, A5316; 1:4000 dilution). After incubation, membranes were rinsed three times with PBS (5 minutes each) and incubated for 45 minutes with secondary antibodies conjugated to IRDye® 680 or IRDye® 800 dyes (LI-COR, 1:2000 dilution). Following three additional 5-minute PBS washes, the membranes were scanned and analyzed using the Odyssey IR Imaging System (LI-COR) and Image Studio™ Software (Version 5.2).

### Statistical analysis

The statistical tests and number of replicates for each experiment are indicated in the figure legends. Data are presented as mean ± standard deviation (SD). The interaction terms of the two-way ANOVA factors (load, age, and genotype) are reported in the corresponding graph. Statistical analysis and graphical design were performed using GraphPad Prism 10.4.1 (GraphPad Software, Inc., La Jolla, CA, USA). Group means were compared using a two-sided Student’s t-test or two-way repeated measures ANOVA, as appropriate. Normality and homogeneity of variances were assessed using the D’Agostino and Pearson test and Shapiro-Wilk test, respectively. Pairwise multiple comparisons were performed with *p*-values adjusted by the Holm–Sidak method. Statistical significance was defined as *p*<0.05.
